# The proprotein convertase furin is required to maintain viability of alveolar rhabdomyosarcoma cells

**DOI:** 10.18632/oncotarget.11648

**Published:** 2016-08-27

**Authors:** Patricia Jaaks, Gianmarco Meier, Nagjie Alijaj, Eva Brack, Peter Bode, Ewa Koscielniak, Marco Wachtel, Beat W. Schäfer, Michele Bernasconi

**Affiliations:** ^1^ Department of Oncology and Children's Research Center, University Children's Hospital Zurich, Zurich, Switzerland; ^2^ Department of Surgical Pathology, University Hospital Zurich, Zurich, Switzerland; ^3^ Department of Oncology/Hematology/Immunology, Olgahospital, Klinikum Stuttgart, Stuttgart, Germany

**Keywords:** furin, proprotein convertases, rhabdomyosarcoma, apoptosis, IGF1R

## Abstract

Rhabdomyosarcoma (RMS) is the most common soft tissue sarcoma in children. Success of current therapies is still limited and outcome is particularly poor for metastatic alveolar rhabdomyosarcoma (aRMS). We previously identified the proprotein convertase furin as potential target for specific drug delivery with RMS-homing peptides. Furin is a protease that converts inactive precursor proteins into bioactive proteins and peptides. In this study, we investigate the biological role of furin in aRMS progression *in vitro* and *in vivo*. Furin expression was confirmed in over 86% RMS biopsies in a tissue microarray (n=89). Inducible furin silencing *in vitro* led to significant impairment of cell viability and proliferation in all investigated aRMS cell lines, but not in MRC5 fibroblasts. Furthermore, the aRMS cell lines Rh3 and Rh4 revealed to be very sensitive to furin silencing, undergoing caspase-dependent cell death. Notably, furin silencing *in vivo* led to complete remission of established Rh4 tumors and to delayed growth in Rh30 tumors. Taken together, these findings identify furin as an important factor for aRMS progression and survival. Thus, we propose furin as a novel therapeutic target for treatment of aRMS.

## INTRODUCTION

Rhabdomyosarcoma (RMS) is the most common pediatric soft tissue sarcoma, representing 5-8% of all childhood cancers. RMS is subdivided in two main histological subgroups, embryonal (eRMS) and alveolar (aRMS) RMS. aRMS is more aggressive and is associated with a poorer prognosis with a 5-year survival of 48% [1]. Around 80% of aRMS tumors harbor the chimeric transcription factor PAX3/7-FOXO1 inducing a specific gene expression signature [2, 3]. A dominant role of PAX3/7-FOXO1 as oncogenic driver has been suggested [4, 5]. Despite the dependence of aRMS tumors on PAX3/7-FOXO1 expression, transcription factors in general represent a challenging target.

Thus, many efforts have been made to characterize key pathways driving RMS progression in order to identify targets for novel therapeutic approaches. The insulin-like growth factor 1 receptor (IGF1R) is one among them. IGF1R is a transcriptional target of PAX3-FOXO1 [6] and increased levels of the receptor correlate with poorer outcome in aRMS patients [7]. The ligand of IGF1R, the insulin-like growth factor 2 (IGF-2), is overexpressed in RMS [8] and acts as an autocrine mitogen [9]. Therefore, the IGF1R signaling pathway is a promising target to treat aRMS tumors. Different approaches to disrupt IGF1R signaling in RMS have been investigated: IGF1R anti-sense RNA [10], IGF1R-specific blocking antibodies [11] and the selective IGF1R inhibitor NVP-AEW541 [12].

Proteolytic processing of precursor proteins by proprotein convertases (PCs) produces a large variety of bioactive proteins, such as growth factors, receptors, enzymes and cell-adhesion molecules. PCs are calcium-dependent serine proteases and seven of the nine family members (PC1, PC2, furin, PC4, PC5, paired basic amino acid cleaving enzyme 4 (PACE4) and PC7) process proproteins at basic residues. The other two, SK-1 and PCSK9, cleave after non-basic residues [13]. Enhanced activity of PCs has been associated with pathological conditions like Alzheimer's disease [14] and correlates with increased malignancy of certain cancer types such as prostate cancer, colon carcinoma or small cell lung carcinoma [15–17].

Furin was the first identified PC and acts within the constitutive secretory pathway [18]. It processes many cancer-related proteins like IGF1R [16], the vascular endothelial growth factor C (VEGF-C) [19] or the membrane-type 1 matrix metalloprotease (MT1-MMP) [20]. Aberrant furin expression is associated with neoplasias like head and neck cancer, breast, lung or colon cancers [21–24]. Upregulation of furin expression and translocation to the plasma membrane under hypoxic conditions are considered to favor invasiveness of cancer cells through enhanced proteolytic activation of MT1-MMP and TGFβ [25, 26]. Thus, furin is a key activator of proproteins involved in cancer progression and represents a promising target to improve cancer treatment. Furin is endogenously inhibited by its own prodomain that is cleaved in a two-step autocatalytic activation process and the use of the prodomain as furin inhibitor has been proposed [27, 28]. Alternative inhibitory approaches are application of polyarginines [29], nanobodies [30] or α1-antitrypsin Portland (PDX) [31]. However, most if not all proposed inhibitors lack selectivity for furin and are still in early developmental phases.

We previously identified specific RMS homing peptides that bind to the proprotein convertase (PC) furin [32]. Here, we investigate in detail the role of furin in aRMS progression *in vitro* and *in vivo*. We use inducible expression of furin specific shRNA to decrease furin expression and activity. Furin silencing resulted in impaired cell viability, caspase-dependent apoptosis and regression of tumors. We further found that furin is expressed in most patient biopsies. Therefore, we propose the proprotein convertases furin as novel target for aRMS therapy.

## RESULTS

### Validation of inducible furin silencing in alveolar RMS cells

**Figure 1:**
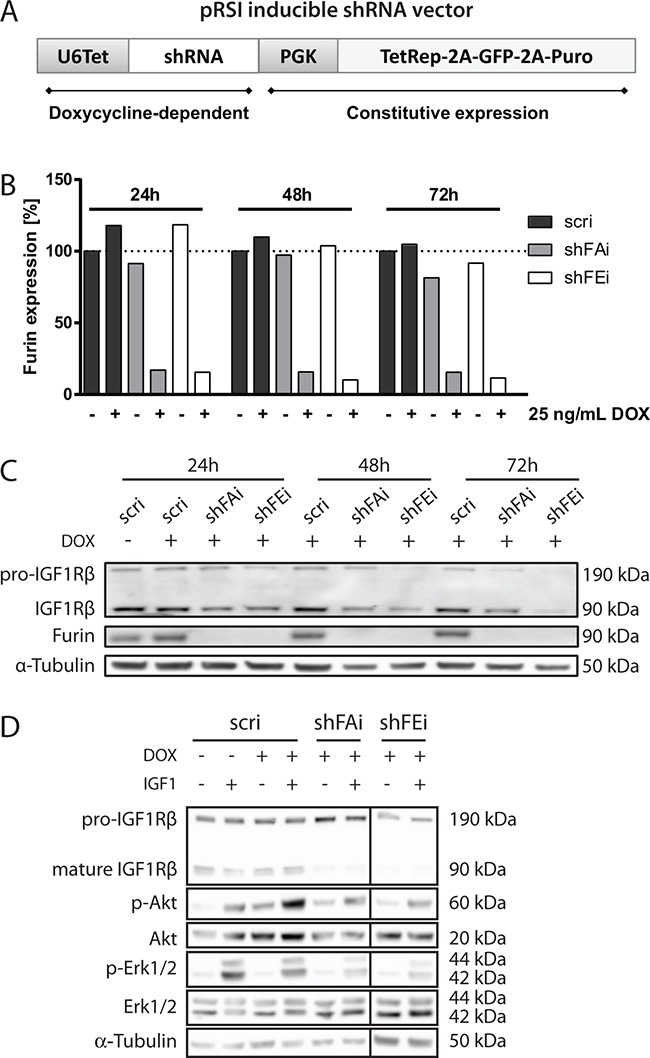
Inducible silencing of furin in alveolar RMS cells **A.** Schematic overview of the pRSI vector for inducible shRNA expression. Tet repressor, GFP and puromycin resistance under control of a phosphoglycerate kinase 1 (PGK) promoter are constitutively expressed. The tetracycline dependent promoter (U6Tet) controls expression of shRNA. Rh30 cells transduced with pRSI coding for control (scri) or furin (shFAi, shFEi) shRNAs were treated with 25 ng/mL doxycycline (DOX). **B.** Furin mRNA levels were determined in Rh30 cells by qRT-PCR at different time points after DOX addition. Expression levels relative to GAPDH and normalized to non-treated control (scri) cells are shown. The experiments were performed in duplicates. **C.** Protein expression of furin and its substrate IGF1Rβ were examined in Rh30 cells by immunoblotting 24h, 48h and 72h post-induction. One representative experiment is shown. **D.** Rh3 cells were stimulated for 10 min. with 50 ng/mL IGF1 48h post induction of furin silencing and phosphorylation of IGF signaling pathway mediators Akt and Erk1/2 was examined by immunoblotting. One representative experiment is shown.

In order to simulate specific furin inhibition we generated stable aRMS cell lines with tetracycline-dependent expression of furin shRNA (shFAi and shFEi) or control shRNA (scri) (Figure 1A). Furin silencing was assessed 24-72h after doxycycline (DOX) treatment. Upon DOX treatment furin mRNA was reduced to around 15% in Rh30 cells (Figure 1B) and to 6.5-25% in other aRMS cell lines (Supplementary Figure S1A). Subsequently, we analyzed furin protein levels in Rh30 cells by immunoblotting und could confirm that furin protein levels were strongly reduced upon DOX treatment (Figure 1C). To monitor furin activity we analyzed the maturation of a known furin substrate, IGF1Rβ. Decrease of furin led to clear reduction of mature IGF1Rβ, indicating that furin activity is indeed lowered upon silencing of furin (Figure 1C). mRNA levels of other PCs, examined by qRT-PCR after 48h of treatment with 25 ng/mL DOX, were unaffected suggesting that furin silencing was specific and was not compensated for by increased mRNA expression of other PCs (Supplementary Figure S1B). In conclusion, we generated and validated aRMS cell lines with functional furin silencing that is inducible by application of DOX. Furthermore, effects of furin silencing on IGF1R processing and downstream Akt activation upon stimulation with IGF1 could be demonstrated in Rh3 cells (Figure 1D) and to a lesser extent in Rh4 cells (Supplementary Figure S1C). This suggests that activation of the IGF signaling pathway is furin dependent at least in a subset of aRMS cells.

### Furin silencing decreases cell viability and proliferation

**Figure 2:**
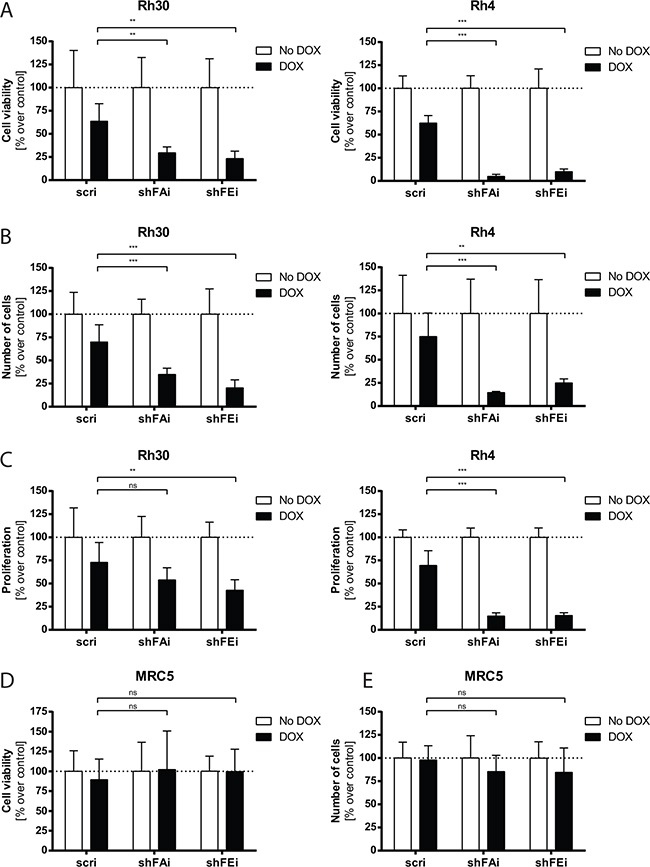
Cell viability and proliferation of aRMS cells are decreased upon furin silencing Rh30, Rh4 and MRC5 cells were treated with 25 ng/mL DOX for 96h and analyzed. **A.** Cell viability in Rh30 and Rh4 cells was determined with a WST-1 assay. **B.** The number of Rh30 and Rh4 cells was quantified by staining with crystal violet. **C.** Proliferation in Rh30 and Rh4 cells was measured by incorporation of BrdU. **D.** Cell viability in MRC5 cells was determined with a WST-1 assay. **E.** The number of MRC5 cells was quantified by staining with crystal violet. All data represent mean ±SD of three independent experiments. *P<0.05, **P<0.01, ***P<0.005, two-way ANOVA.

Furin processes a variety of substrates, e.g. IGF1R, that support cancer cell viability and proliferation. Thus, we induced furin silencing in four different aRMS cell lines, Rh30, Rh4, CW9019 and Rh3, and assessed cell viability. Cell viability was clearly decreased, as compared to non-treated cells (Figure 2A - Rh30: 27%, Rh4: 7%; Supplementary Figure S2A - CW9019: 48%, Rh3: 19%). The number of viable adherent cells was reduced up to 5-fold compared to non-treated cells (Figure 2B - Rh30: 23%, Rh4: 20%; Supplementary Figure S2B - CW9019: 54%, Rh3: 23%). Furthermore, proliferation, examined by BrdU incorporation, was much lower in furin silenced Rh30 and Rh4 cells than in cells expressing control shRNA and went down to 48% and 15%, respectively (Figure 2C). We also noted a slight toxic effect upon expression of the control shRNA (scri) in all aRMS cell lines (Figure 2A–2C). Since DOX application alone had no effect on viability of aRMS wt cells (data not shown), we assume that this slight effect may be due to high shRNA expression after DOX treatment. Furthermore, we investigated furin silencing in MRC5 fibroblast cells and found that induced silencing of furin had no significant impact on viability of MRC5 fibroblast cells (Figures 2D and 2E). Thus, furin activity seems to be a critical factor in viability and proliferation of aRMS cells, whereby a decrease of furin does not alter cell viability of normal fibroblasts.

### Reduced furin activity results in caspase-dependent cell death involving mitochondria

**Figure 3:**
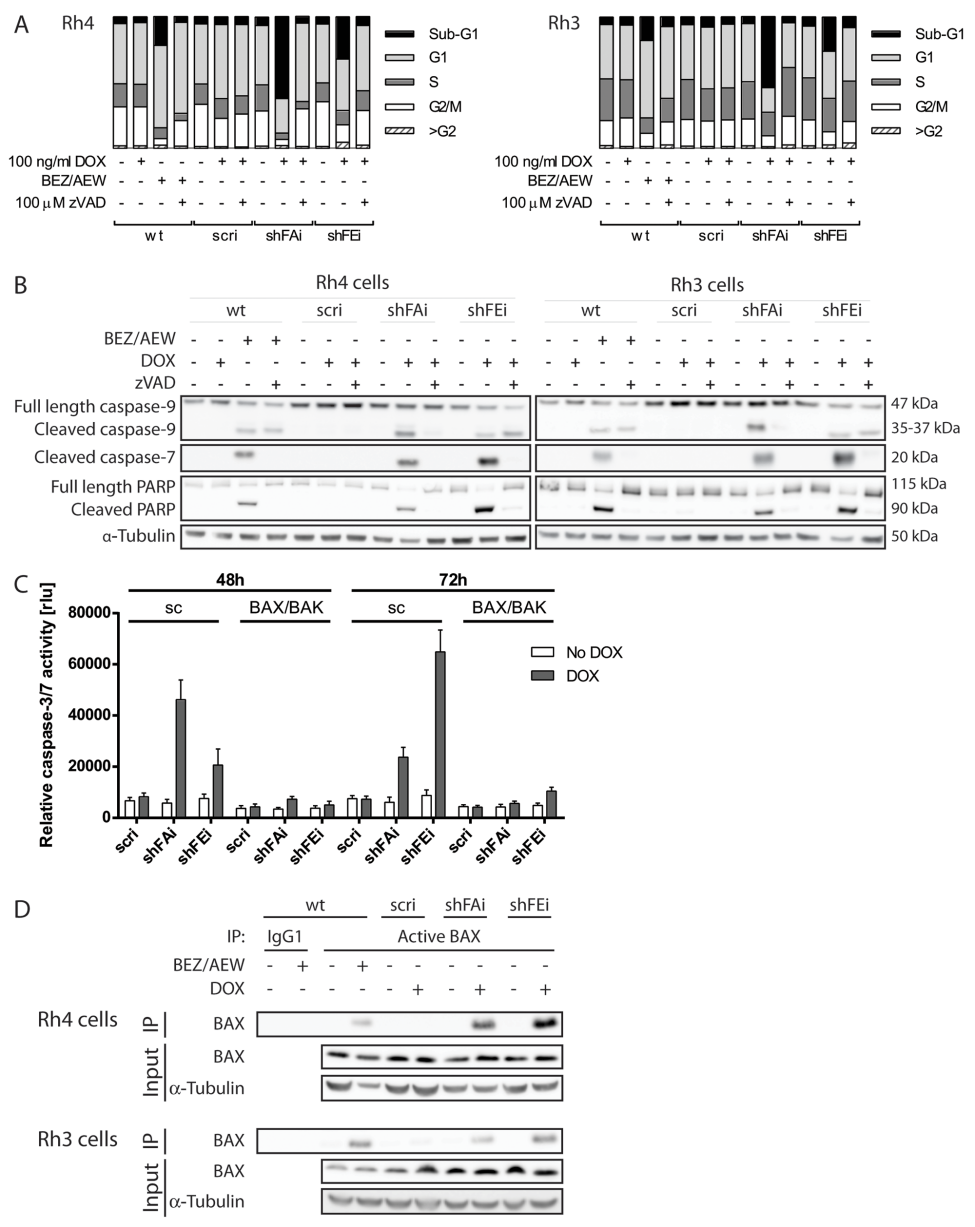
Cell death induction upon furin silencing is caspase dependent and involves mitochondria **A.** Caspase inhibition restores normal cell cycle distribution. Rh4 and Rh3 cells were treated with 100 ng/mL DOX for 72h in the presence or absence of 100 μM zVAD, fixed and stained with propidium iodide and cell cycle distribution was studied by FACS. **B.** Inhibition of caspase activity rescues cleavage of caspase-7 and PARP, but not caspase-9 cleavage. Furin silencing was induced in Rh4 (left panel) and Rh3 (right panel) cells by addition of 100 ng/mL DOX in the presence or absence of zVAD, total protein was extracted 72h post induction, and cleavage of caspase-9 and -7 and PARP was analyzed by immunoblotting. Wt cells were treated with 275 nM BEZ235 and 1 μM AEW451 for 24h as positive control (A-B, D). **C.** Effector caspase activation is mitochondria dependent. Rh4 cells with a stable BAX and BAK (BAX/BAK) or control (sc) knock out were generated using a lentiviral CRISPR/Cas9 based approach. Knock out cells were stably transduced with inducible furin silencing constructs. Cells were treated with 100 ng/mL DOX for 48 and 72h and caspase-3/7 activity was analyzed by a caspase specific luminescent assay. Data represent mean ±SD of three independent experiments. *P<0.05, **P<0.01, ***P<0.005, two-way ANOVA. **D.** Apoptosis induction is dependent on BAX activation. Rh4 and Rh3 cells were treated with 100 ng/mL DOX for 48h (scri, shFAi) or 72h (shFEi) and active BAX was immunoprecipitated using a conformation-specific antibody.

In our initial investigation of aRMS cell viability upon furin silencing we observed that reduced furin activity not only impaired cell viability and proliferation of the cells, but also led to morphological changes and cell death in Rh4 and Rh3 cells. Therefore, we examined the cell cycle distribution of Rh4 and Rh3 cells 72h after DOX treatment and found a clear increase of cells in sub-G1 phase (Figure 3A - shFAi: 54-62%, shFEi: 26-32%). As a positive control for apoptosis we treated aRMS cells with a combination of 1 μM of the IGF1R-inhibitor AEW451 (AEW) and 275 nM of the dual PI3K/mTOR inhibitor BEZ235 (BEZ) for 24h, conditions that induce apoptosis in aRMS cell lines [33]. As expected, treatment with BEZ/AEW led to an increase of cells in sub-G1 phase (Figure 3A - Rh4: 22%, Rh3: 18%). To assess the contribution of caspases in cell death induction we treated the cells with 100 μM of the pan-caspase inhibitor zVAD. Cell cycle profiles of furin silenced or BEZ/AEW treated cells were restored in the presence of zVAD similar to non-treated levels, indicating that induced cell death is caspase dependent (Figure 3A).

To further elucidate the mechanism underlying cell death, we analyzed cleavage of caspase-7 and -9 and PARP by immunoblotting. In Rh4 and Rh3 cells, both caspases as well as PARP were cleaved upon furin silencing and BEZ/AEW treatment, confirming apoptosis induction. Cleavage of the effector caspase-7 and of PARP could be almost completely prevented in the presence of zVAD, whereby inhibition of caspase-9 cleavage was incomplete (Figure 3B). Caspase activity of the caspases-3 and -7 was elevated 5 to 7-fold upon furin silencing in Rh4 and Rh3 (Figure 3C and data not shown), but not in Rh30 or CW9019 cells (data not shown). This suggests that in some aRMS cells furin silencing mainly inhibits proliferation and viability, whereas in other aRMS cell lines a decrease in furin activity additionally induces caspase-dependent cell death. Therefore, application of zVAD to Rh4 cells upon induction of furin silencing only partially rescued cell viability as assessed by measuring cell viability and number of attached cells (Supplementary Figure S3A and S3B). In this case caspase inhibition prevented apoptosis, but did not decrease anti-proliferative processes.

To examine the involvement of mitochondria in apoptosis induced by furin silencing we generated Rh4 cells with a stable double knock out of BAX and BAK (BAX/BAK) or control (sc) knock out. Levels of BAX and BAK proteins in heterogeneous cell pools were investigated by immunoblotting. For both proteins knock out efficiency was almost complete (Supplementary Figure S4). Rh4 cells with BAX/BAK or control knock out were then transduced with inducible furin shRNA as described above. In control knock out Rh4 caspase-3/7 activity peaked at 48h for shFAi and at 72h for shFEi and was elevated 7.9-fold or 7.4-fold when compared to non-treated cells. In contrast, caspase-3/7 activity levels were increased only minimally upon furin silencing in the absence of BAX and BAK (Figure 3C). Furin silencing in Rh4 and Rh3 cells further stimulated activation of BAX, as was shown by immunoprecipitation of BAX with active conformation-specific antibodies (Figure 3D). These results suggest that furin silencing leads to caspase-dependent apoptosis that relies on mitochondria pore formation through BAX and BAK.

### Furin silencing results in remission of Rh4 xenografts *in vivo*

**Figure 4:**
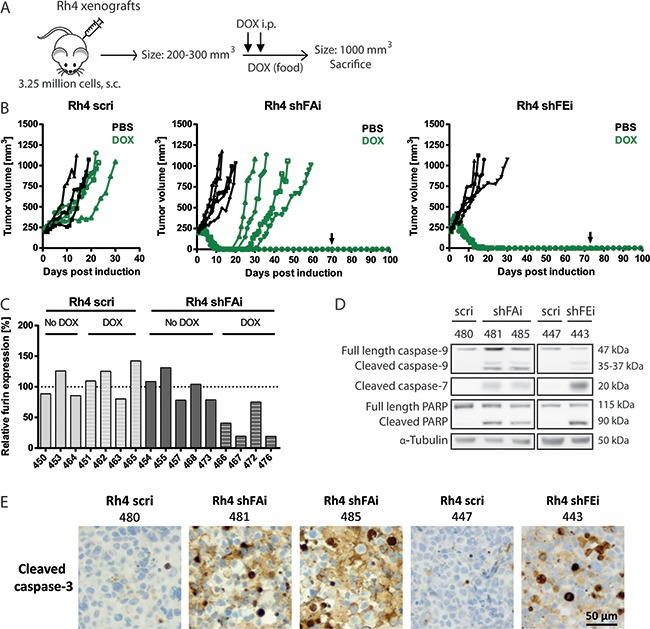
Induction of furin silencing results in remission of Rh4 tumors *in vivo* **A.** Schematic overview of Rh4 xenograft model. 3-5 NOD/SCID mice per group were engrafted s.c. with 3.25 million Rh4 cells. Expression of shRNA was induced through administration of DOX (i.p. injection of 53 mg/kg DOX on two consecutive days combined with DOX-supplemented food) once a tumor size of 200-300 mm^3^ was reached. Mice were sacrificed upon a tumor size of 1000 mm^3^. **B.** Tumor growth over time as monitored by caliper measurements in Rh4 xenografts. Arrows indicate that mice were put back on normal food approximately 60 days post onset of remission. **C.** Furin mRNA levels in Rh4 tumors. Tumor tissue was collected at sacrifice, total RNA isolated and furin mRNA levels analyzed by qRT-PCR. **D–E.** Apoptotic markers in Rh4 tumors after 5 days of DOX treatment. D) Caspase-9, cleaved caspase-7 and PARP levels were examined by Western blot. E) Levels of cleaved caspase-3 were analyzed by immunohistochemistry.

Reduced furin activity decreased cell viability and proliferation in Rh4 cells and further induced caspase-dependent apoptosis *in vitro*. To examine the role of furin in Rh4 tumor growth we engrafted 3.25 million cells harboring either inducible furin shRNA (shFAi and shFEi) or control shRNA (scri). In order to simulate a potential therapy aiming at abrogating furin activity in established tumors, mice were treated with DOX once tumors reached a size of 200-300 mm^3^ (Figure 4A). Mice were sacrificed upon reaching a tumor size of 1000 mm^3^. PBS-treated tumors took on average 16-17 days to attain this size (Figure 4B -scri: 17 days; shFAi: 16.6 days; shFEi: 16.5 days). Rh4 scri tumors treated with DOX showed slightly decelerated tumor growth and took on average 25 days to reach a size of 1000 mm^3^. In contrast, Rh4 shFAi and shFEi tumors went into full remission after 10-15 days. 4 out of 5 Rh4 shFAi tumors relapsed 9-20 days later, whereby the last tumor of this group never relapsed, not even after DOX withdrawal 60 days post remission. None of the Rh4 shFEi DOX-treated mice relapsed (Figure 4B). Assessment of furin mRNA by qRT-PCR revealed that only half of Rh4 shFAi DOX-treated relapsed tumors maintained low levels of furin. The other two tumors showed higher furin mRNA levels (41% and 75% over control treated Rh4 shFAi tumors), indicating a possible outgrowth of cells with ineffective furin silencing (Figure 4C). To further investigate the mechanisms leading to remission of Rh4 shFAi and shFEi tumors upon DOX administration, we sacrificed few mice 5 days after initial administration of DOX. Decreased furin mRNA levels were confirmed by qRT-PCR (Supplementary Figure S5 - shFAi: around 30%; shFEi: 15%). Total protein was extracted from tumor tissue and protein levels of different cell death markers were examined by immunoblotting. The three tumors with lower furin levels displayed elevated levels of cleaved caspase-9 and -7 and cleaved PARP as compared to Rh4 scri tumors (Figure 4D). Furthermore, Rh4 shFEi, but especially shFAi tumors showed high levels of cleaved caspase-3 after 5 days of DOX treatment (Figure 4E). Expression of scri in control tumors did not lead to activation of this effector caspase.

Thus, we could show that induced silencing of furin leads to full remission of Rh4 tumors *in vivo*. This is most likely achieved through initiation of apoptotic processes, as shown by activation of key caspases. Beside reactivation of furin expression, other, yet undetermined, processes might lead to relapse of tumors and should be further investigated.

### Tumor growth of Rh30 xenografts *in vivo* is delayed after furin silencing

**Figure 5:**
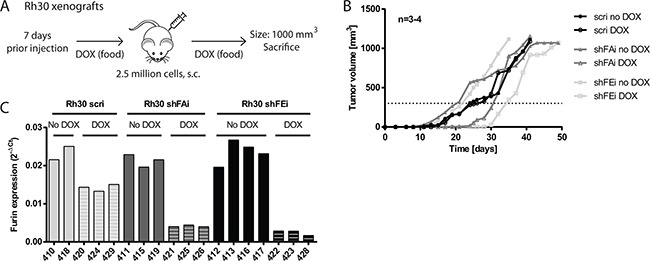
Decrease of furin activity delays tumor growth of Rh30 xenografts *in vivo* **A.** Schematic overview of Rh30 xenograft model. 3-4 NOD/Scid mice per group were fed with or without DOX-supplemented food starting 7 days prior engraftment. Mice were then engrafted s.c. with 2.5 million Rh30 cells. Tumor growth was monitored over time and mice were sacrificed once a tumor reached a size of 1000 mm^3^. **B.** Absence of furin activity delays initial tumor growth. Tumor growth rate was monitored by caliper measurements. Data represent mean tumor size of 3-4 mice per group. **C.** Furin expression is reduced upon induction of furin specific shRNA. Tumor tissue was collected upon sacrifice of mice, total RNA isolated and furin mRNA levels analyzed by qRT-PCR. Expression levels relative to HMBS are shown.

Induction of furin silencing diminished proliferation and cell viability of Rh30 cells *in vitro*. To determine the impact of furin activity on Rh30 tumor growth *in vivo* we engrafted 2.5 million Rh30 cells with inducible furin shRNA and shRNA expression was induced by administration of DOX-supplemented food 7 days prior engraftment of tumor cells (Figure 5A). Control groups were fed normal food. Tumor growth was monitored over time through caliper measurements and mice were sacrificed once tumors reached a size of 1000 mm^3^. Rh30 scri tumor growth was similar with or without DOX supplement and tumors reached a size of 300 mm^3^ after 25 days. Conversely, early tumor growth was clearly delayed in Rh30 shFAi and shFEi tumors in the presence of DOX and tumors took approximately 50% more time to reach 300 mm^3^ (Figure 5B, shFAi - no DOX: 21 days, DOX: 31 days; shFEi - no DOX: 22 days, DOX: 34 days). After reaching a size of 300 mm^3^ most Rh30 tumors grew at the same pace independent of the genetic background. To investigate furin silencing efficiency we extracted RNA from tumor tissue at sacrifice and evaluated furin mRNA levels by qRT-PCR. Furin mRNA levels of Rh30 shFAi or shFEi tumors with DOX as compared to respective tumors without DOX were on average 15% and 11%, respectively (Figure 5C), confirming effective furin silencing. Despite having low furin levels, once furin silenced Rh30 tumors exceeded a size of approximately 300 mm^3^, they grew almost as fast as control tumors. However, a clear delay in early tumor growth could be observed, suggesting that furin activity is important in early phases of Rh30 tumor growth *in vivo*.

### Most RMS biopsies express furin

**Figure 6:**
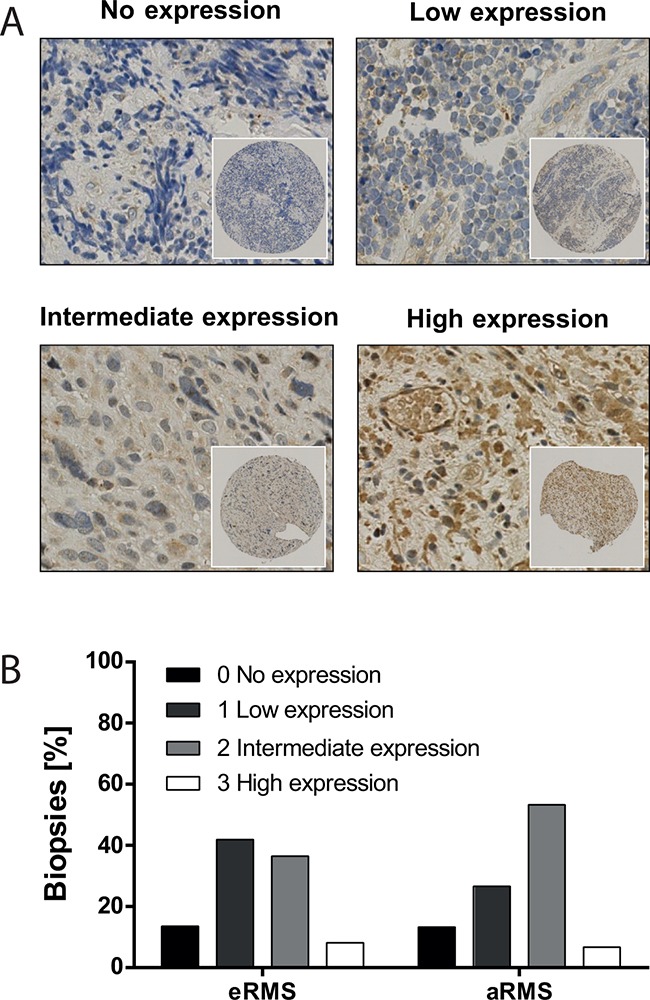
Furin is expressed in most RMS biopsies A patient-derived tissue microarray was immunohistochemically stained for furin (n=89). Patients were grouped according to no expression (n=12), low (n=35), intermediate (n=35) and high (n=7) furin expression. **A.** Representative pictures of biopsies from all four categories. **B.** Distribution of furin expression intensity based on RMS subtype. Furin expression levels were analyzed for 74 eRMS and 15 aRMS biopsies.

To evaluate the potential of furin targeted therapies in patients we examined furin protein expression in 89 RMS biopsies (74 eRMS, 15 aRMS). A TMA was stained for furin and expression was graded according to overall intensity of the staining in tumor cells (Figure 6A). We found that, independent of the subtype, over 86% of RMS tumors expressed low to high amounts of furin (Figure 6B), whereby most tumors showed low to intermediate expression. A correlation between the level of furin protein in tumors and event-free or overall survival of the patients could not be confirmed (data not shown).

## DISCUSSION

This study establishes for the first time a link between furin activity and rhabdomyosarcoma progression both *in vitro* and *in vivo*. Silencing of furin led to significantly decreased cell viability and induction of caspase-dependent apoptosis *in vitro*. Furthermore, depletion of furin activity *in vivo* resulted in regression or delayed tumor growth of aRMS tumors.

Furin is known to process many growth factors and their receptors, like IGF-1 and 2, IGF1R or transforming growth factor β (TGFβ) [34, 35]. Accordingly, silencing of furin led to significant reduction of cell viability and proliferation rate in all investigated aRMS cell lines. Furin silencing had no effect on MRC5 fibroblast viability, suggesting that aRMS, but not normal cells are dependent on furin activity. A subset of the aRMS cell lines tested was particularly sensitive to furin silencing and underwent BAX/BAK- and caspase-9-dependent apoptosis. This indicates that furin silencing primarily triggers the intrinsic apoptotic pathway. Others have shown that disruption of the IGF1R and/or PI3K/mTOR pathways leads to apoptosis in Rh3 and Rh4 cell lines [12, 33]. In this study we found evidence that activation of the IGF signaling pathway is dependent on furin activity. We therefore hypothesize that incomplete activation of growth factor-dependent pathways is the principal cause of cell death induced upon furin silencing in sensitive cell lines. It remains to be investigated which proapototic BH3-only proteins are involved, but treatment of Rh3 and Rh4 cells with a combination of the IGF1R inhibitor AEW541 and the dual PI3K/mTOR inhibitor BEZ235 results in BMF-dependent apoptosis [33]. Other proapototic BH3-only proteins linked to growth factor deprivation are BAD, BIM and PUMA. For instance, phosphorylation of BAD through active protein kinase Akt results in binding and sequestration by 14-3-3 scaffold proteins [36, 37]. Thus, lower levels of activated Akt due to decreased IGF signaling upon furin silencing might promote release of BAD and mitochondria pore formation. Further investigations will be required to elucidate the exact mechanism of cell death after silencing of furin.

To examine the impact of furin silencing on aRMS tumor growth *in vivo* we engrafted two aRMS cell lines, Rh30 and the sensitive Rh4. In Rh30 xenografts, upon furin silencing, we observed delay of early tumor growth, suggesting that furin cleaves one or more precursor proteins that are crucial for early steps of aRMS tumor growth after xenotransplantation.

A signaling pathway likely to be involved is the VEGF signaling axis. Gee et al. confirmed expression of VEGF, VEGFR1, which is a transcriptional target of PAX3-FOXO1 [38], and VEGFR2 in aRMS cell lines and were the first to propose a role of VEGF signaling in RMS growth [39]. Processing of precursor VEGF-C and VEGF-D through furin allows their binding to VEGFR2 and induction of vasculogenesis and angiogenesis [19, 40–42]. Additionally, only furin-processed VEGF-C induces tumorigenesis in mice [19], highlighting the importance of PC activity in the VEGF signaling axis. Therefore, reduced VEGF-C and VEGF-D processing upon furin silencing might suppress proper formation of angiogenic and lymphangiogenic vessels inside the new tumors and decrease VEGFR2-dependent aRMS tumor cell growth.

When furin was silenced in established Rh4 tumors, regression was observed. Apoptosis was induced, as confirmed by activation of caspases-9, -3 and -7 and PARP cleavage. Four of the Rh4 shFAi tumors relapsed and furin re-expression was found in two of them. Since we used a heterogeneous cell pool, it is likely that outgrowth of a subset of non-silenced cells caused the relapse. Further investigations will be required to uncover the underlying mechanisms of tumor regrowth.

We found furin expression in over 86% of tested aRMS and eRMS biopsies, but no significant correlation could be confirmed between furin expression and event-free or overall survival. It is possible that other risk-factors interacted with the analysis of furin influence on survival, or that furin expression alone is not a good marker for its role in cancerogenesis, but rather its subcellular localization and activity are. Our results are in line with other studies demonstrating the role of furin and other PCs for cancer cell survival [15, 28, 43]. Thus, PCs are emerging targets for the treatment of cancer and development of specific inhibitors will be of central importance. Levesque et al. recently reported the improvement of peptide-based specific PACE4 inhibitors for treatment of prostate cancer [44, 45] and Zhu et al. described the first furin-specific inhibitory nanobodies [30]. Despite their great clinical potential, specific inhibitors of furin and furin-like PCs are still in early phases of development. Besides, the safety of such inhibitors will have to be closely examined as most PCs are widely expressed in human tissues. Nevertheless, disruption of furin activity has been used successfully to enhance an anti-tumor immune response in patients with advanced cancers. Safety and efficient immune response of an autologous tumor cell vaccine (FANG) that combines furin shRNA and expression of GM-CSF were confirmed and benefits of treatment were observed from phase I trials in Ewing sarcoma and hepatocellular carcinoma patients [46–48]. Additionally, increased presence of furin on the cell surface of tumor cells could be used for targeted delivery of chemotherapeutics, as we could show previously that furin is the target receptor for RMS-homing peptides [32]. In conclusion, our data clearly validate furin as a novel target in aRMS and we predict that the majority of pediatric RMS patients would benefit from inhibition of furin activity, either alone or in the context of immune therapy.

## MATERIALS AND METHODS

### Cell lines and cell culture

The cell lines Rh4, Rh30 (Peter Houghton, St. Jude Children's Hospital, Memphis, TN), Rh3 (Susan Ragsdale, St. Jude Children's Hospital, Memphis, TN), CW9019 (Soledad Gallego, Hospital Universitari Vall d'Hebron, Barcelona, Spain), MRC5 and HEK293T (ATCC, LGC Promochem) were cultured in DMEM (Sigma-Aldrich), supplemented with 100 U/mL penicillin/streptomycin, 2 mM L-glutamine, and 10% FBS (Life Technologies) in 5% CO_2_ at 37 °C. aRMS cell lines were tested and authenticated by cell line typing analysis (STR profiling) on 2011/2014 and positively matched [49].

### Production of lentiviral particles and transduction of cells

Production of lentiviral particles and transduction of cells are described in Supplementary Material and Methods.

### qRT-PCR

Total RNA was extracted from cells or tumor tissue using the Qiagen RNeasy Kit (Qiagen, Hombrechtikon, Switzerland) and reverse-transcribed using oligo (dT) primers and Omniscript reverse transcriptase (Qiagen). qRT-PCR pas performed for furin (Hs00965485_g1), PC1 (Hs01026107_m1), PC2 (Hs0015992_m1), PC4 (Hs00399493_m1), PC5 (Hs00196400_m1), PACE4 (Hs00159844_m1), PC7 (Hs00237114_m1), S1P (Hs00921626_m1) and PCSK9 (Hs00545399_m1) employing TaqMan gene expression master mix (all Life Technologies). Cycle threshold (C_T_) values were normalized to GAPDH (Hs02758991_g1) or HMBS (Hs00609296_g1) for cells or tumor tissue, respectively. Relative expression levels were calculated using the ΔΔC_T_ method.

### Immunoblotting

Total protein extracts from cells or tumor tissue were separated using 4-12% Bis-Tris SDS-PAGE gels (Life Technologies) and transferred to nitrocellulose membranes (PROTAN; Schleicher & Schuell). Membranes were blocked with 5% milk powder in TBS/0.05% Tween and subsequently incubated with primary antibodies overnight at 4°C. After washing in TBS, membranes were incubated with IgG horseradish peroxidase (HRP)-linked antibody for 1h at RT. Proteins were detected using ECL detection reagent (Fisher Scientific) after washing in TBS.

### IGF1 stimulation

RMS cells were treated with 25 ng/mL DOX for a total of 48h. After 24h cells were put on starvation medium (DMEM, 0.2% FBS). Cells were stimulated for 10 min with 50 ng/mL IGF1 (ab73455, Abcam). Cells were washed with ice-cold PBS and snap frozen in liquid nitrogen. Whole cell lysates were prepared as described above and IGF1Rβ, Akt, phosphorylated Akt, Erk1/2 and phosphorylated Erk1/2 were analyzed by immunoblotting.

### Antibodies

Antibodies are described in Supplementary Material and Methods.

### Cell proliferation and viability assays

Cells were seeded in a 96-well format in 100 μL medium. To assess cell viability 10 μL WST-1 reagent (Roche) were added. After 30-60 min incubation at 37°C in the dark absorbance was measured and recorded as ΔOD_440-640_. Background values were subtracted. To determine their number, cells were fixed in 4% paraformaldehyde for 10 min at RT and stained in 0.05% crystal violet (Sigma-Aldrich) for 20 min at RT. Excess crystal violet was removed and cells air dried. Dye was dissolved in 100% methanol and absorbance was measured at OD_594_. Cell proliferation was determined through BrdU incorporation (Cell Proliferation ELISA, BrdU (Chemiluminescent), Roche) according to manufacturer's instructions. Cells were labeled with BrdU for 2h at 37°C, fixed and stained for 1h with anti-BrdU-POD solution. Chemiluminescent substrate was added and luminescence measured.

### Caspase 3/7 activity

Cells were seeded in a 384-well format in 20 μL medium. At the desired time point 10 μL Caspase Glo^®^ 3/7 Substrate (Promega) were added and plates vigorously shaken for 1 min. After 30 min incubation at RT in the dark luminescence was measured.

### Cell cycle distribution

One million cells were collected and fixed in ice cold 70% ethanol at -20°C for at least 2h. Fixed cells were washed in PBS and resuspended in propidium iodide (PI) solution (20 μg/mL PI and 200 μg/mL RNase A (Sigma-Aldrich) in PBS/0.1% Triton X-100). PI signal was assessed by FACS. Cell cycle distribution was analyzed with FlowJo software, version 10 (TreeStar).

### Immunoprecipitation of active BAX

For detection of active BAX, cells were lysed in CHAPS lysis buffer (10 mM HEPES (pH 7.4), 150 mM NaCl, 1% CHAPS) as previously described [50]. Briefly, 1000 μg protein were incubated overnight at 4°C with 5 μg mouse anti-BAX antibody (6A7, Sigma) followed by addition of 10 μL Dynabeads Protein G (Invitrogen) and further incubation for 2h at 4°C. Dynabeads were washed with lysis buffer and BAX levels were analyzed by immunoblotting using rabbit anti-BAX (1:1000; 5023, Cell Signaling).

### Xenograft studies

To study *in vivo* growth 2.5×10^6^ Rh30 or 3.25×10^6^ Rh4 cells were engrafted subcutaneously in 6-week-old NOD/SCID IL2rg^−/−^ mice (female, 20-25 g; Charles River). In Rh30 xenografts doxycycline (DOX)-supplemented food (625 mg/kg, Synergy Health, Däniken, Switzerland) was given one week prior engraftment. DOX administration in Rh4 xenografts was started upon a tumor size of 200-300 mm^3^ through intraperitoneal (i.p.) injection of 0.053 mg DOX/g body weight on two consecutive days and continuous DOX-supplemented food. Control treated mice were injected with PBS. Body weight and tumor growth were monitored over time. Tumor volumes were determined by measuring two diameters (d1, d2) in right angles using a digital caliper (V = (4/3) π r^3^; r = (d1 + d2)/4). Upon reaching a tumor size of 1000 mm^3^ mice were sacrificed, tumor tissue was harvested and snap frozen for RNA and protein extraction.

### Immunohistochemistry

Sections (3-μm thick) of formalin-fixed, paraffin-embedded tissue were mounted on glass slides (SuperFrost Plus; Menzel), deparaffinised, rehydrated and stained with hematoxylin and eosin (H&E). Immunohistochemistry was performed on Leica BondMax instruments using Refine HRP-Kits (Leica DS9800; Leica Microsystems Newcastle, Ltd.). Dewaxed and pretreated (Epitop Retrieval Buffer 2, Leica Biosystems, Muttenz, Switzerland; 60 min, 100°C) paraffin slides were incubated with anti-cleaved caspase-3 antibody (1:500; #9661, Cell Signaling).

### Tissue microarray

A tissue microarray with 248 cores from 124 RMS tumors (17 aRMS with known *FOXO1* gene rearrangements and 107 eRMS) was constructed. Tumors used were collected at the University Hospital Zurich, Switzerland and at the Kiel Pediatric Tumor Registry, Germany. Immunohistochemistry was performed essentially as described above. Paraffin slides were dewaxed, pretreated (Epitop Retrieval Buffer 2, Leica Biosystems;10 min, 95°C) and incubated with furin antibody (1:3000; Ab28547, Abcam). Overall staining was graded according to intensity and assigned to four different groups (no expression, low, intermediate or high expression). Tissue microarrays were analyzed double-blinded.

## SUPPLEMENTARY MATERIALS METHODS AND FIGURES


