# Supplementary material for: The proprotein convertase furin is required to maintain viability of alveolar rhabdomyosarcoma cells
Source: Oncotarget. 2016 Aug 27;7(47):76743–55. doi: 10.18632/oncotarget.11648 (PMC5363546; doi:10.18632/oncotarget.11648)
Supplement: Supplementary file 1 [file oncotarget-07-76743-s001.pdf]

The proprotein convertase furin is required to maintain viability of alveolar rhabdomyosarcoma cells

SUPPLEMENTARY MATERIALS AND METHODS

Ethics statement

All animal experiments were approved and monitored by the veterinary office of the Canton of Zurich according to the Swiss Federal Law.

Statistical analysis

Statistical analysis was performed using GraphPad Prism. Data are expressed as mean ± standard deviation (SD). Statistical significance was tested for multiple comparisons with analysis of variance (ANOVA). The differences were considered to be significant if $p<0.05$.

Production of lentiviral particles and transduction of cells

Third generation lentiviral packaging plasmids and vectors containing shRNA (scrambled (scri) or targeted against furin (target sequence shFAi: CCTGTCCCTCTAAAGCAATAA; target sequence shFEi: CCACATGACTACTCCGCAGAT)) under a U6-tet promoter as well as reporter genes GFP and puromycine resistance under PGK promoter were purchased from Cellecta (Cellecta Inc. Mountain View, USA). Lentiviral particles were generated by calcium phosphate-based transfection of HEK293T cells. Transducing units per mL (TU/mL) were determined through titration of lentiviral particles on Rh4 cells and analysis of % GFP positive cells by fluorescent activated cell sorting (FACS) on a FACSCanto II (BD Bioscience, Missisagua, Canada) 48h post transduction. TU/mL were determined using the formula TU/mL= (F*C*D)/V (F=frequency of GFP positive cells; C=number of cells; D=virus dilution; V=volume of inoculum). Cells were transduced with a multiplicity of infection (MOI) of 5. Transduction was performed by addition of required amount of lentiviral particles in DMEM in the presence of 10 µg/mL polybrene (Sigma-Aldrich) followed by centrifugation (32 °C, 1h, 800 g). Medium was replaced with fresh medium 4h post transduction start and efficiency of transduction was determined by analysis of GFP-positive cells by FACS 7-10 days post transduction. Transduction efficiency was over 98% for all cell lines.

To generate Rh4 cells with double knock out for BAX and BAK a CRISPR/Cas9-based approach was chosen. Vectors based on the LentiCrispr v1 vector (Addgene) were modified to carry a TagBFP for selection and delivered packaged in lentiviral particles. Two different sgRNAs for each target gene were evaluated individually for their knock down efficiency by Western blot and the two best sequences were then co-delivered to achieve a double knock out (Rh4 BAX/BAK; sgRNA BAX: CGAGTGTCTCAAGCGCATCG; sgRNA BAK: ACGGCAGCTCGCCATCATCG). A scrambled sgRNA sequence was used as control (Rh4 sc; sgRNA sc: GCACTACCAGAGCTAACTCA).

Antibodies

The following antibodies were used: furin MON-152 (1:750, ALX-803-017-R100, Alexis Corporation), anti-IGF1Rβ (9750S, Cell Signaling), anti-caspase-9 (9502, Cell Signaling), anti-cleaved caspase-7 (9491, Cell Signaling), anti-PARP (9542, Cell Signaling), anti-Akt (9272, Cell Signaling), anti-phospho-Akt (Ser437, 9018, Cell Signaling), anti-Erk1/2 (9102, Cell Signaling), anti-phospho-Erk1/2 (4370, Cell Signaling) and anti-α-tubulin (1:5000, A5316, Sigma-Aldrich). All Cell Signaling antibodies were used in a dilution of 1:1000.

SUPPLEMENTARY FIGURES

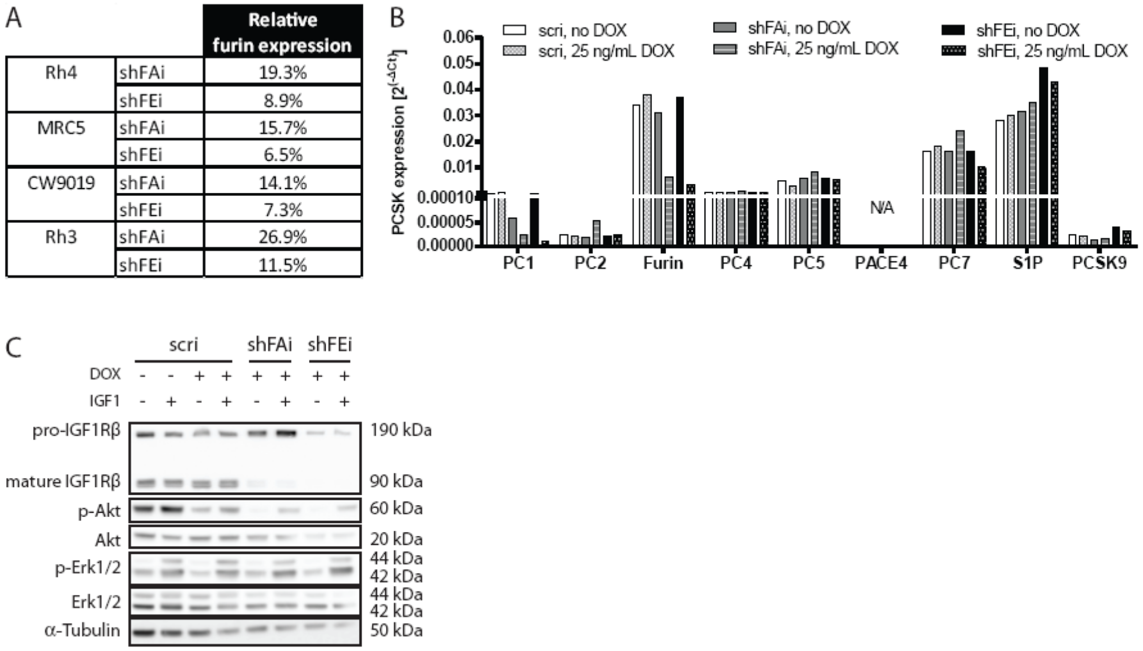

Supplementary Figure S1: Validation of furin silencing in other cell lines and expression of other PCSKs. A. Furin silencing efficiency. Rh4, MRC5, CW9019 and Rh3 cells were treated with 25 ng/mL DOX for 48h, total mRNA was isolated and furin mRNA levels were studied by qRT-PCR. Data represent relative furin expression over non-treated scri control. **B.** Expression of other PCSKs upon furin silencing. Furin silencing was induced in Rh30 cells through addition of 25 ng/mL DOX, total mRNA was extracted 48h post induction and mRNA levels of other proprotein convertase family members were analyzed by qRT-PCR. **C.** Rh4 cells were stimulated for 10 min. with 50 ng/mL IGF1 48h post induction of furin silencing and phosphorylation of IGF signaling pathway mediators Akt and Erk1/2 was examined by immunoblotting. One representative experiment is shown.

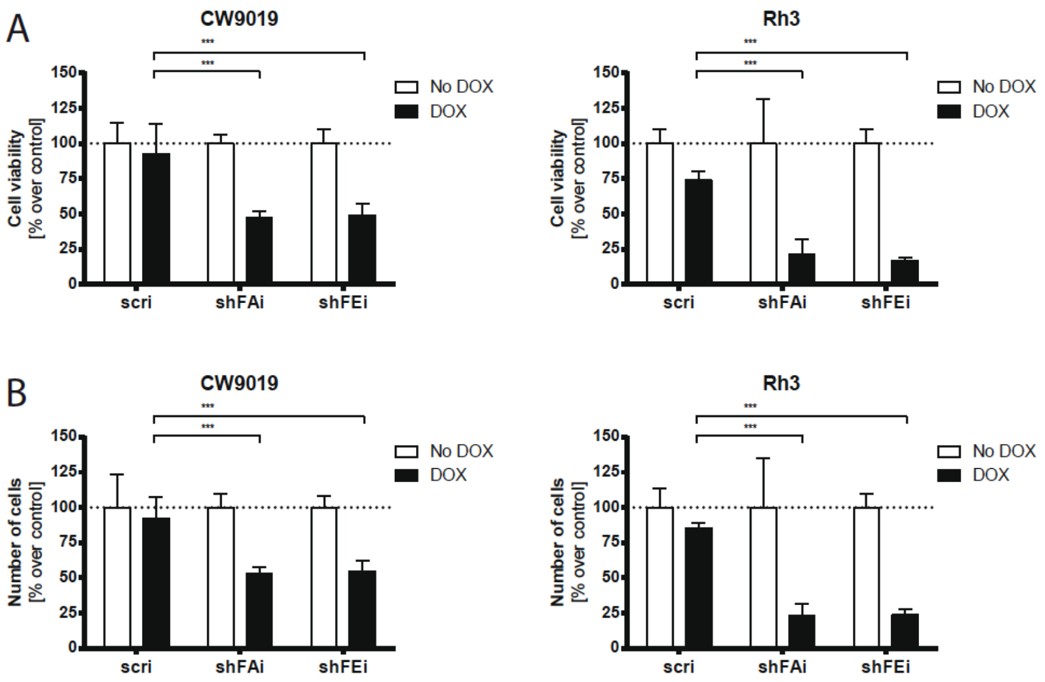

Supplementary Figure S2: Cell viability in other aRMS cell lines is affected upon loss of furin activity. CW9019 and Rh3 cells were treated with 25 ng/mL DOX for 96h. **A.** Cell viability was analyzed in a WST-1 assay. **B.** The number of cells was determined by staining with crystal violet. All data represent mean ±SD of three independent experiments. *P<0.05, **P<0.01, ***P<0.005, two-way ANOVA.

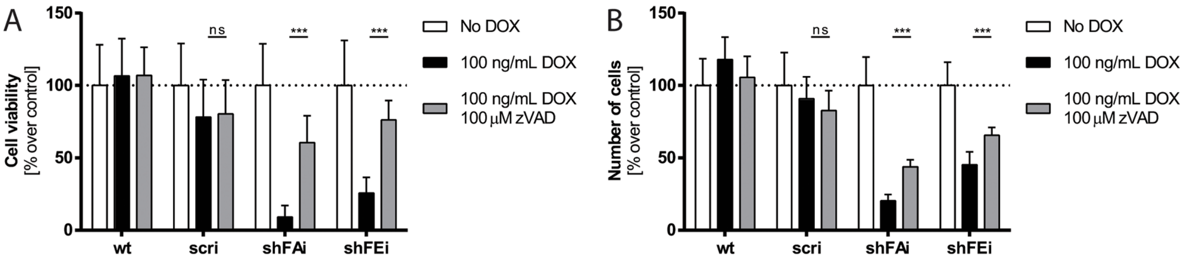

Supplementary Figure S3: Caspase inhibition partially rescues cell viability in Rh4 cells. Rh4 cells were treated with 100 ng/mL DOX for 72h in the presence or absence of 100 µM zVAD. **A.** Cell viability was examined in a WST-1 assay. **B.** The number of cells was analyzed by staining with crystal violet. Data represent mean ±SD of three independent experiments. *P<0.05, **P<0.01, ***P<0.005, two-way ANOVA.

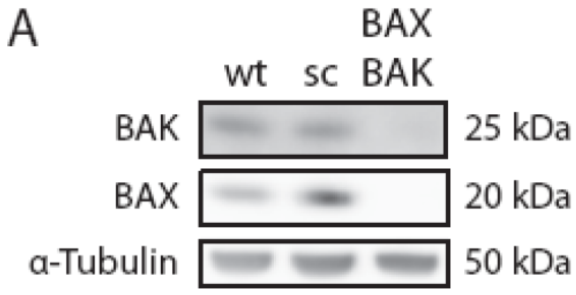

Supplementary Figure S4: Validation of BAX/BAK knock out in Rh4 cells. Total protein of Rh4 wt, sc and BAX/BAK cells was extracted and BAX and BAK protein levels were analyzed at protein level by Western blot.

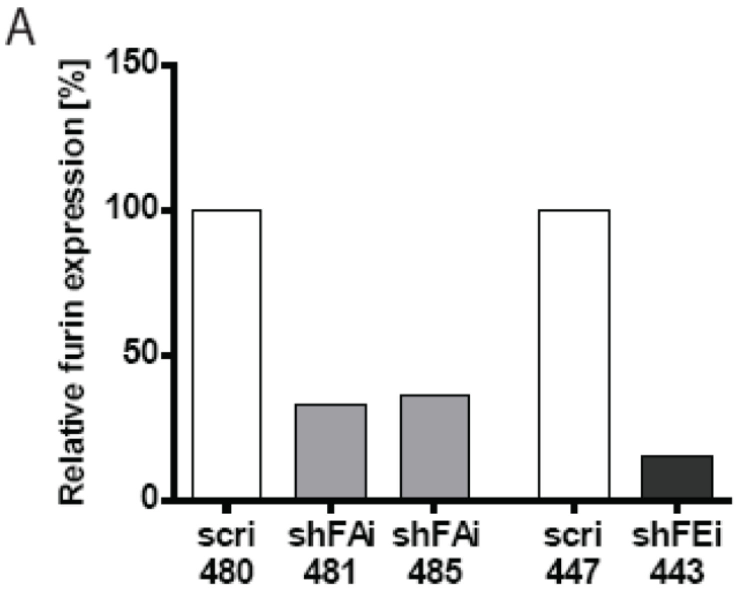

Supplementary Figure S5: Furin silencing in Rh4 xenografts 5 days post induction. NOD/Scid mice were engrafted s.c. with 3.25 million Rh4 cells. Expression of shRNA was induced through administration of doxycycline (i.p. injection of 53 mg/kg doxycycline on two consecutive days combined with doxycycline supplemented food) once a tumor size of 250-300 mm^3 was reached. Mice were sacrificed 5 days post induction. Total mRNA was extracted from tumor tissue and furin mRNA levels analyzed by qRT-PCR. Depicted are expression levels over HMBS.
